# Changes in Cardiovascular Health during Young Adulthood and Subclinical Atherosclerosis in Middle Age: The CARDIA Study

**DOI:** 10.5334/gh.1179

**Published:** 2023-03-17

**Authors:** Xiaomin Ye, Zhenyu Xiong, Jiaying Li, Yifen Lin, Peihan Xie, Xiangbin Zhong, Rihua Huang, Xiaodong Zhuang, Xinxue Liao

**Affiliations:** 1 The First affiliated hospital, Sun Yat-Sen University, CN; 2 NHC Key Laboratory of Assisted Circulation, Sun Yat-sen University, CN; 3 Guangdong Provincial Geriatrics Institute, Guangdong Provincial People’s Hospital, Guangdong Academy of Medical Sciences, CN; 4 Center for Information Technology & Statistics, the First Affiliated Hospital, Sun Yat-Sen University, CN

**Keywords:** Ideal cardiovascular health, coronary artery calcification, carotid intima-media thickness, young adults

## Abstract

**Background and aims:**

The benefits of reaching ideal cardiovascular health (CVH) are well known, but it is unclear whether positive CVH changes from young adulthood to middle age reduce subclinical atherosclerosis risk. This study examined associations of changes in CVH from young adulthood to middle age and CVH in young adulthood with subclinical atherosclerosis.

**Methods:**

Data was analyzed from the Coronary Artery Risk Development in Young Adults (CARDIA) study. CVH was examined at years 0 and 20 using Life Simple 7 metrics from AHA guideline. Coronary artery calcium (CAC) was identified at years 20 and 25. Carotid intima-media thickness (IMT) was identified at year 20.

**Results:**

Among 2,935 participants (56.2% women, 46.7% black), the change of CVH score was –1.26 (2.13). For per 1-unit increase in CVH at baseline, the adjusted odds ratios (ORs) of presence of CAC and IMT were 0.81 (95% CI 0.78, 0.86) and 0.85 (95% CI 0.76, 0.94), respectively. For per 1-unit increase in CVH changes, the adjusted ORs of CAC and IMT were 0.86 (95% CI 0.82, 0.90) and 0.81 (95% CI 0.73, 0.90). Compared with stable moderate CVH, improvement from moderate to high was associated with a lower risk of CAC (0.64 [95% CI 0.43, 0.96]), while retrogression from moderate to low was associated with a higher risk of CAC (1.45 [95% CI 1.19, 1.76]).

**Conclusions:**

Positive changes of CVH during young adulthood are associated with negative subclinical atherosclerosis risk in middle age, indicating the importance of reaching an ideal cardiovascular health status through young adulthood.

## Introduction

Life’s Simple 7 metrics, a concept of cardiovascular health (CVH) proposed by the American Heart Association (AHA), is assessed with four metabolic risk factors (body mass index, blood pressure, blood glucose, total cholesterol) and three healthy lifestyles (diet, physical activity, smoking) [1]. They have been shown to be important cardiovascular risk factors, participants of which mostly were chosen from the elder population in current studies [2,3,4]. However, not enough attention was paid to the younger population or subclinical cardiovascular events, such as coronary artery calcium (CAC) and intima-media thickness (IMT), broadly accepted as marker and maker of cardiovascular events [5,6,7,8]. Considering components of cardiovascular health are time-varying variables, associations of changes in CVH and subclinical atherosclerosis remain uncertain. To find out association between CVH scores and CAC is critical in preventing atherosclerosis risk in young adulthood.

This study used the Coronary Artery Risk Development in Young Adults (CARDIA) study with the aim of quantifying the association between CVH and its changes through young adulthood with the incidence of CAC and IMT in midlife, and we hypothesized that positive CVH changes from young adulthood to middle age would reduce subclinical atherosclerosis risk.

## Patients and methods

### Data and material disclosure statement

Data documentation for CARDIA is publicly available online (cardia.dopm.uab.edu.). Data used in this article are available on reasonable request from the CARDIA coordinating Center.

### Study population

Details of the CARDIA study design and examinations have been reported previously [9]. The CARDIA study is a longitudinal cohort study which began in 1985–1986 (Y0). A cohort sample of 5,115 healthy participants were recruited at four US sites: Birmingham, Alabama; Chicago, Illinois; Minneapolis, Minnesota; and Oakland, California, and were balanced for gender, race, age (18–24 years and 25–30 years), and educational level (high school or less education and greater than high school education). Seven follow-up examinations of the cohort were conducted at years 2, 5, 7, 15, 20 (Y20), and 25. At each study, all participants provided written informed consent and the institutional review boards at each study site and coordinating center have granted approval annually for all examinations.

The flow chart of this study is presented in supplementary figure 3. Of the initial 5,115 recruited participants at baseline, 1,587 did not return at Y20 and 593 had missing CVH data or covariates data, leaving 2,935 participants included in the basic analysis. For analysis of CAC, 123 participants who had not taken CT scan at either Y20 or Y25 were excluded, while for analysis of IMT 244 participants who had not taken carotid ultrasound at Y20 were excluded, leaving 2,812 participants included in the analysis of CAC and 2,691 participants included in the analysis of IMT.

### Cardiovascular health

CVH, consistent with Life’s Simple 7 according to AHA recommendation, consists of four components of metabolic risk: blood pressure, blood glucose, total cholesterol, and body mass index, and three components of lifestyle: smoking, physical activity, and diet. All metrics were measured at baseline and Y20, obtained by trained and certified technicians. Blood pressure was measured three times after five minutes rest at seated and recorded as the average of second and third-time measurement. Participants were asked to fast for 12 hours before venous blood was drawn for blood glucose and total cholesterol analysis. Body mass index was calculated as weight in kilograms divided by height in meters, squared. Cigarette smoking history was self-reported. Physical activity scoring was assessed from a physical activity questionnaire and calculated with the frequency and duration of 13 kinds of activities. Dietary intakes were assessed from interviewer-administrated, quantitative food history [10]. Medication use for controlling blood pressure, cholesterol, or glucose was self-reported. Covariates such as age, gender, race, education level, and history of disease were self-reported by questionnaire.

CVH scores were calculated according to prior studies [11,12,13] (Table 1). According to prior study [11], we chose 40% as a cut-off point for physical activity. Participants with top 40% physical activity were given two points, and participants with lowest 20% were given zero points, the rest were given one point. Dietary intake measure in CARDIA is most useful for ranking individuals based on one-month consumption rather than absolutely quantifying food intake [14]. Therefore, we adjusted the dietary score based on traditional AHA standard and calculated separately for each sex with four food sources: potassium(mg), calcium(mg), fiber(g), and saturated fat(g). A higher score was assigned to participants with higher intake of potassium, calcium, and fiber and a lower intake of saturated fat (High = 5 to low = 1). Four scores summed up to a total score ranging from 4 to 20. The highest 40% get two points. This method was proven to be in concordance with other dietary eating measure patterns and the traditional AHA standard [11,12]. Based on CVH scores, participants were assigned to 3 classes: high (12–14 points), moderate (8–11 points), and low (0–7 points). CVH scores at baseline subtracted from those at Y20 are the change of CVH scores from baseline to Y20.

**Table 1:** Definition of Cardiovascular health.


	CARDIOVASCULAR HEALTH		

	IDEAL = 2 POINTS	MODERATE = 1 POINT	POOR = 0 POINT

BMI, kg/m^2^	<25	25–29.9	>30

Smoking	Never, quit over 1year	Former, quit less than 1 year	Current

Diet	Top 40%	Second 40%	Lowest 20%

Blood pressure, mmHg	<120/80 without medication	Systolic 120–139 Diastolic 80–89 Treated <120/80	>140/90

Total cholesterol, mg/dL	<200 without medication	200–239 Treated <200	>=240

Fasting glucose, mg/dL	<100 without medication	100–125 Treated <100	>=126

Physical activity	Top 40%	Second 40%	Lowest 20%


### Coronary artery calcium

The extent of CAC was quantified at years 20 and 25 using a standardized protocol [15]. At Y20, two sequential CT scans were provided using either a cardiac-gated electron beam CT scanner or a multidetector CT system. At Y25, only a single CT scan was performed, using a 64-channel multidetector CT system. Prior study suggested that two CT scans were comparable [16]. Computed tomography images were read by experienced technicians in a central reading center. Total CAC score was obtained by Agatston method [17], summing up all lesions within a given artery and across all arteries (left main, left anterior descending, left circumflex, and right coronary artery). A CAC score over zero at either Y20 or Y25 was defined as the presence of CAC.

### Intima-media thickness

Images of distal common carotid artery, the carotid bulb, and the proximal internal carotid artery on both sides were obtained by high-resolution B-mode ultrasonography using a standardized protocol at Y20. Intima-media thickness was calculated from the average of the mean intima-media thickness for the internal, bulb, and common carotid near and far walls of the right and left sides [18]. Carotid intima-media thickness over one was defined as a presence of the thickening of carotid intima-media thickness, which is abnormal IMT.

### Statistical analyses

Continuous data are expressed as means ± SD and categorical variables are presented as counts and proportions. Multivariable adjusted logistic regression analyses were conducted to estimate the association of baseline CVH and changes and presence of CAC and abnormal IMT. Multivariable adjusted linear regression analyses were used to estimate the association between changes of CVH and IMT. We adjusted for demographic variables (age at baseline, gender, race, and education), current drinker, history of hypertension, history of diabetes. We also performed subgroup analysis based on gender, race, alcohol use, history of hypertension, history of diabetes. We further adjusted for family income at Y5 to measure the socio-economic status affect.

A 2-sided P value of <0.05 was considered statistically significant. All analyses were performed using SPSS version 26 and Stata SE version 15.

## Results

### Characteristic of participants

**Figure 1:**
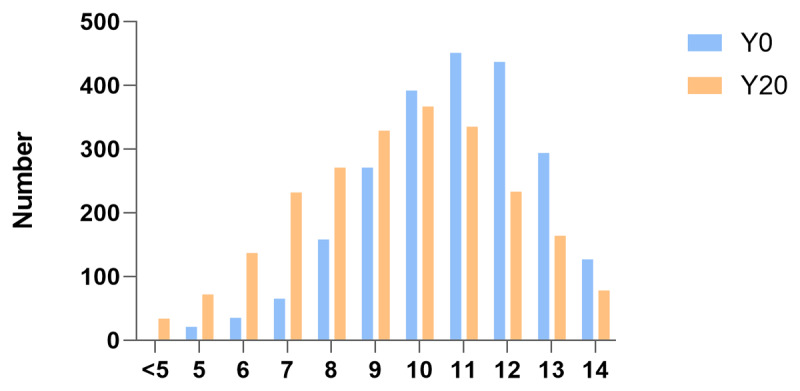
Distribution of Cardiovascular health scores.

The characteristics of the participants at Y0 and Y20 were presented at Table 2. Of 2,935 participants at baseline, 43.8% were male, 46.7% were black, and the average (SD) age was 25 (3.59) years. The proportion of participants with high CVH class dropped from 38.3% at the baseline to 21.8% at Y20, while participants with low CVH class climbed from 5.55% to 21.8%. The incident rate of CAC was 26.5% (744/2,812). The incident rate of abnormal IMT was 3.6% (98/2,691). The average change of CVH scores from baseline to Y20 is –1.26 ± 2.13. The distribution of CVH points and CVH classes at both Y0 and Y20, and also the 20-year changes of both were presented at Figure 1 and Supplementary figure 4–6. Most of the CVH scores of participants were in the 10–12 range at Y0, while the majority were in the 9–11 range at Y20, showing the overall downward trend of CVH from Y0 to Y20.

**Table 2:** Characteristics of Participants at Baseline and Y20 (n = 2935).


	Y0	Y20

Gender		

Male	1285 (43.8%)	

Female	1650 (56.2%)	

Race		

Black	1372 (46.7%)	

White	1563 (53.3%)	

Education	13.9 (1.99)	14.7 (1.91)

Age, years	25.0 (3.59)	45.2 (3.57)

Alcohol consumption		

Never	396 (13.5%)	643 (21.9%)

Current/Ever	2539 (86.5%)	2292 (78.1%)

Smoking status		

Never	750 (25.6%)	563 (19.2%)

Ever	168 (5.72%)	67 (2.28%)

Current	2017 (68.7%)	2305 (78.5%)

BMI, kg/m^2^	24.2 (4.59)	29.2 (6.43)

SBP, mmHg	110 (10.7)	116 (14.5)

DBP, mmHg	68.5 (9.35)	72.0 (11.1)

Total cholesterol, mg/dL	177 (32.9)	185 (34.7)

Fasting glucose, mg/dL	82.1 (12.2)	97.9 (26.4)

Physical activity	422 (300)	338 (277)

Medicine for lowering blood pressure		

No	2872 (98.0%)	2430 (82.9%)

Yes	60 (2.05%)	501 (17.1%)

Medicine for lowering high cholesterol		

No	569 (99.8%)	2663 (91.0%)

Yes	1 (0.18%)	264 (9.02%)

Medicine for lowering blood glucose		

No	566 (99.3%)	2791 (95.3%)

Yes	4 (0.70%)	138 (4.71%)

CVH score	10.8 (1.94)	9.50 (2.41)

CVH Class		

Low	163 (5.55%)	641 (21.8%)

Moderate	1649 (56.2%)	1654 (56.4%)

High	1123 (38.3%)	640 (21.8%)

Change of CVH score	–1.26 (2.13)	

Prevalence of CAC	744/2812 (26.5%)	

Prevalence of IMT	98/2691 (3.6%)	


*Abbreviation*: BMI = body mass index, SBP = systolic blood pressure, DBP = diastolic blood pressure, CVH = Cardiovascular health, CAC = Coronary artery calcification, IMT = intima-media thickness.

### Association between CVH and CAC

The association between CVH and incidence of CAC was presented in Table 3. After adjustment by age, gender, race, education, current drinker, history of hypertension, and history of diabetes, the odds ratio (OR) is 0.81 (95% CI 0.78, 0.86) for per 1 point increase in CVH at baseline. For the further-adjusted baseline CVH score, the OR is 0.86 (95% CI 0.82, 0.90) for per 1 point increase in CVH changes. An inverse association was also found between changes of CVH scores with incidence of CAC (P_trend_ < 0.001) (Supplementary Table 1).

**Table 3:** Baseline and Changes of Cardiovascular health with Prevalence of CAC (n = 2812).


	MODEL 1		MODEL 2		MODEL 3*	
		
	OR (95%CI)	P-VALUE	OR (95%CI)	P-VALUE	OR (95%CI)	P-VALUE

Baseline						

Per 1 point increase	0.80 (0.77, 0.83)	<0.001	0.80 (0.76, 0.84)	<0.001	0.81 (0.78, 0.86)	<0.001

Changes of CVH						

Per 1 point increase	0.95 (0.91, 0.99)	0.007	0.94 (0.90, 0.98)	0.005	0.86 (0.82, 0.90)	<0.001


*Abbreviation*: CAC = Coronary artery calcification, OR = Odds ratio, CVH = Cardiovascular health. Model 1: Unadjusted. Model 2: Adjusted by age, gender, race, education. Model 3: Adjusted by age, gender, race, education, current drinker, history of hypertension, history of diabetes. * In analysis of changes of CVH, Model 3 was adjusted both by baseline CVH score.

In order to assess the specific risk of CAC in different situations, we separated the baseline CVH class into low, moderate, and high classes to see whether the change of CVH class altered the risk of CAC (Table 4). For the moderate class at baseline compared to the stable moderate class, participants who climbed to high class at Y20 had a lower risk of CAC (Adjusted OR 0.55, 95% CI 0.35, 0.89), while those who dropped to low class at Y20 had a higher risk of CAC (Adjusted OR 1.71, 95% CI 1.32, 2.23). Compared to stable high class, the high-to-low class had a higher risk of CAC (Adjusted OR 3.48, 95% CI 1.80, 6.73) and the high-to-moderate class had no significant different risk of CAC (Adjusted OR 1.18, 95% CI 0.81, 1.72). For low class at baseline, significant different risk of CAC was not observed between participants with stable low class and low-to-moderate/high class (Adjusted OR 0.77, 95% CI 0.36, 1.65).

**Table 4:** Changes from different baseline CVH Class with Prevalence of CAC (n = 2812).


	N/N	MODEL 1		MODEL 2		MODEL 3	
		
		OR (95%CI)	P-VALUE	OR (95%CI)	P-VALUE	OR (95%CI)	P-VALUE

Change from low class	65/151						

Low to Low	46/98	Ref		Ref		Ref	

Low to Moderate/High	19/53	0.63 (0.32, 1.26)	0.190	0.65 (0.31, 1.36)	0.252	0.77 (0.36, 1.65)	0.494

Change from moderate class	483/1584						

Moderate to Moderate	278/973	Ref		Ref		Ref	

Moderate to Low	179/447	1.67 (1.32, 2.11)	<0.001	1.88 (1.46, 2.43)	<0.001	1.71 (1.32, 2.23)	<0.001

Moderate to High	26/164	0.47 (0.30, 0.73)	0.001	0.52 (0.32, 0.82)	0.005	0.55 (0.35, 0.89)	0.014

Change from High class	196/1077						

High to High	69/446	Ref		Ref		Ref	

High to low	23/66	2.92 (1.66, 5.16)	<0.001	3.80 (1.97, 7.32)	<0.001	3.48 (1.80, 6.73)	<0.001

High to moderate	104/565	1.23 (0.88, 1.72)	0.219	1.30 (0.90, 1.87)	0.170	1.18 (0.81, 1.72)	0.397


*Abbreviation*: Ref = Reference, CAC = Coronary artery calcification, OR = Odds ratio, CVH = Cardiovascular health. Model 1: Unadjusted. Model 2: Adjusted by age, gender, race, education. Model 3: Adjusted by age, gender, race, education, current drinker, history of hypertension, history of diabetes.

### Association between CVH and IMT

The association between CVH and incidence of abnormal IMT was presented in Table 5. After adjustment by age, gender, race, education, current drinker, history of hypertension, and history of diabetes, the OR is 0.85 (95% CI 0.76, 0.94) for per 1 point increase in CVH at baseline. For the further adjusted baseline CVH score, the OR is 0.81 (95% CI 0.73, 0.90) for per 1 point increase in CVH changes. An inverse association was also found between changes of CVH scores with incidence of abnormal IMT (P_trend_ < 0.001) (Supplementary Table 2). Linear association was also found between changes of CVH scores with IMT (Adjusted β (se) –0.012 (0.001), p-value < 0.001) (Supplementary Table 3).

**Table 5:** Baseline and Changes of Cardiovascular health with Prevalence of abnormal IMT (n = 2691).


	MODEL 1		MODEL 2		MODEL 3*	
		
	OR (95%CI)	P-VALUE	OR (95%CI)	P-VALUE	OR (95%CI)	P-VALUE

Baseline						

Per 1 point increase	0.76 (0.70, 0.84)	<0.001	0.82 (0.74, 0.91)	<0.001	0.85 (0.76, 0.94)	0.002

Changes of CVH						

Per 1 point increase	0.88 (0.80, 0.97)	0.009	0.89 (0.81, 0.98)	0.020	0.81 (0.73, 0.90)	<0.001


*Abbreviation*: IMT = Intima-media thickness, OR = Odds ratio, CVH = Cardiovascular health. Model 1: Unadjusted. Model 2: Adjusted by age, gender, race, education. Model 3: Adjusted by age, gender, race, education, current drinker, history of hypertension, history of diabetes. * In analysis of changes of CVH, Model 3 was adjusted both by baseline CVH score.

In order to assess the specific risk of abnormal IMT in different situations, we separated baseline the CVH class into low, moderate, and high classes to see whether the change of CVH class altered the risk of abnormal IMT (Supplement Table 4). For the moderate class at baseline, compared to stable moderate class, moderate-to-low class had a higher risk of abnormal IMT (Adjusted OR 2.46, 95% CI 1.46, 4.12) while those in the moderate-to-high class did not observe a significant lower risk of abnormal IMT (Adjusted OR 0.70, 95% CI 0.20, 2.37). Compared to the stable high class, the high-to-low and high-to-moderate classes had no significant different risk of abnormal IMT (Adjusted OR 0.60, 95% CI 0.06, 5.64 and Adjusted OR 0.76, 95% CI 0.24, 2.41). For the low class at baseline, significant different risk of abnormal IMT was not observed between participants with stable low class and low-to-moderate/high class (Adjusted OR 0.13, 95% CI 0.01, 1.17).

### Subgroup Analysis

Subgroup analyses based on gender, race, history of hypertension, history of diabetes, and alcohol use were done separately for CAC and abnormal IMT (Supplementary Figure 1 and Figure 2). No significant difference of risk was found between subgroups. After adjustment for family income at Y5, the association between CVH and incidence of CAC and abnormal IMT remained positive. (Supplementary Table 6).

## Discussion

In this study, our central findings were the association between CVH points (at baseline and changes throughout early adulthood) and risk of subclinical atherosclerosis (CAC and IMT). Improvement on CVH points at baseline and changes by one point both could bring down incidence of CAC and IMT. For participants with moderate CVH class at baseline, the majority of the young population, altering CVH class could benefit in the reduction of midlife risk of subclinical atherosclerosis.

Cardiovascular health in our study contains both metabolic factors and healthy lifestyle factors, as AHA recommended [1]. Comparing prior studies [19,20,21], which only focus on one aspect, our study evaluates cardiovascular health better, from more comprehensive assessment criteria. Prior studies have proven CVH scores at baseline were inversely associated with risk of CAC and IMT in middle age to elderly populations [22,23]. Our study fulfilled the blank area in the young adulthood to middle age population. Since the influence of cardiovascular health continues throughout life, focusing on the younger population helps prevent cardiovascular disease earlier. Neglecting variation, single measurement was considered in previous studies [22,23]. Only one study measured the changes of CVH during youth population, but it did not observe significant association between them, which was opposite to our results [24]. The failure of demonstrating real association in the population may result from its smaller sample and shorter follow-up time. Our study verified the association between CVH changes and subclinical atherosclerosis in a large size, multi-ethnic, long follow-up cohort study, which ensured the high reliability of our results.

The follow-up of the CARDIA study begun in 1985–1986, when young adults in the United States began to change dietary habits because of the high consumption of fast food [25]; therefore, they might have different cardiovascular risk factors from the last generation. Considering cardiovascular risk may be different with the alteration of generations, we use Life Simple 7, a common measurement for cardiovascular risk assessment, to evaluate early subclinical atherosclerosis risk in young adulthood. In this study, we found that lower CVH class or scores in young adulthood was associated with higher risk of CAC in midlife. Moreover, compared to those within the stable CVH class, participants with worsened CVH class in midlife had significantly higher risk of CAC, no matter which CVH class they fell into at baseline. It suggests that we should pay attention to cardiovascular health earlier in young adulthood, and should not relax vigilance even in midlife. In this study, the majority of participants are in the moderate CVH class at baseline. Compared to the stable moderate CVH class, participants might suffer more subclinical atherosclerosis risk at midlife if they neglect healthy lifestyle or metabolic factors in young adulthood and fall down to low class. On the contrary, participants might gain profit in reducing midlife risk with early intervention. For example, a participant can gain two points by quitting smoking or losing weight, and see a reduction in midlife risk of 28% (central figure). Instead of focusing on all aspects, people can choose one aspect to work on based on their situation, which is easier to help achieve the goal of preventing CVD. Young adults can add morning running to their daily routine to get higher scores in both physical activity and BMI aspects if losing weight at the same time. Great social enlightenment has been shown in our study: cardiovascular health in young adulthood is as important as that in midlife. Therefore, more attention should be paid to young adulthood for primary prevention of CVD.

**Figure:**
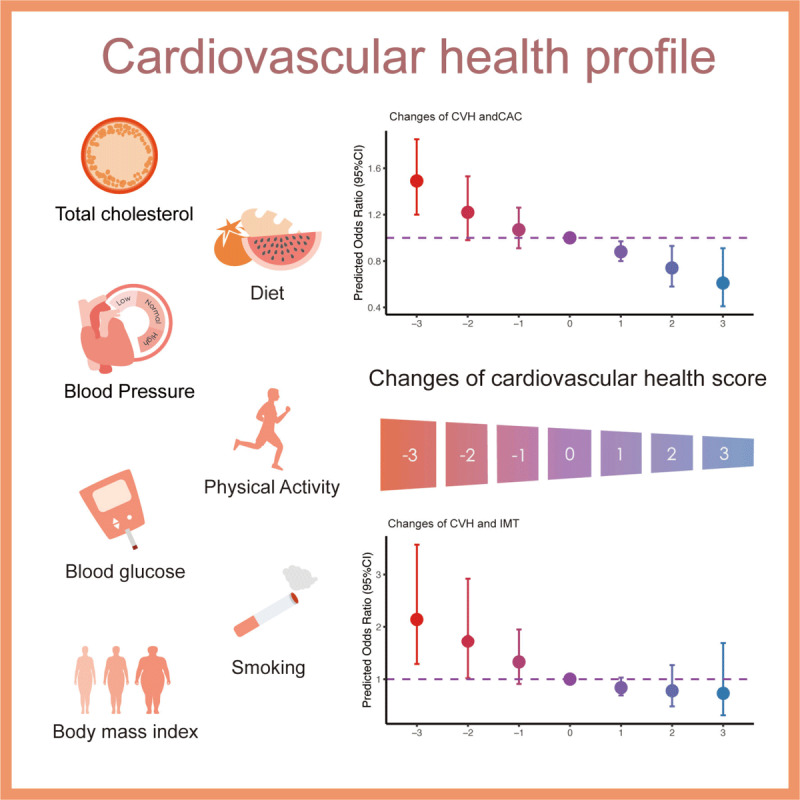


Key strengths of this study include, first, CARDIA study is representative in studying cardiovascular health of young adults for its identity: a multi-center, long follow-up prospective cohort recruiting participants in 18–25 years. Second, our study explored thoroughly the association between baseline and longitudinal change of CVH and subclinical atherosclerosis and presented with reliable results. Third, our study fulfilled the blank area of early cardiovascular risk in the young population and provided a strategy of early intervention for improving cardiovascular health in young adulthood.

Limitations should be considered in our study. First, the measurement of changes in CVH by only considering baseline and Y20 data could cause neglection of variation during the 20-year period. Even though the measurement is crude, positive results were shown in our study, which would be more reliable with increased follow-up measurements. Meanwhile, we are conducting a multiple follow-up cohort about Life Simple 7 to explore the association between its variation and CVD. Second, in real world research, the possibility of residual confounding cannot be ruled out, but we adjusted confounders based on the characteristics of CARDIA study as far as possible. Third, participants lost to follow-up had different baseline characteristics compared to follow-up participants. Potential bias due to different loss to follow-up in younger participants, black male participants, and those with a lower educational attainment may have occurred (Supplementary table 5).

In this study, baseline and change in CVH were associated with the risk of subclinical atherosclerosis in young adulthood-to-middle age population, suggesting the importance of promoting CVH class.

## Additional File

The additional file for this article can be found as follows:

10.5334/gh.1179.s1 Supplementary Materials. Supplementary Tables 1 to 6 and Supplementary Figures 1 to 6.
