# Supplementary material for: Changes in Cardiovascular Health during Young Adulthood and Subclinical Atherosclerosis in Middle Age: The CARDIA Study
Source: Glob Heart. 2023 Mar 17;18(1):14. doi: 10.5334/gh.1179 (PMC10022532; doi:10.5334/gh.1179)
Supplement: Supplementary Materials. — Supplementary Tables 1 to 6 and Supplementary Figures 1 to 6. [file gh-18-1-1179-s1.pdf]

Supplementary Files

Supplementary Materials

Formatted: Centered

Supplementary Table 1. Association between CVH changes and CAC (n=2812)

		Model 1		Model 2		Model 3	
	n/N	OR (95%CI)	p for trend	OR (95%CI)	p for trend	OR (95%CI)	p for trend
Q1 (-4, [<-3])	242/794	Ref	0.018	Ref	0.018	Ref	<0.001
Q2 (-1, [-3, -1])	245/962	0.78 (0.63, 0.96)		0.74 (0.59, 0.93)		0.64 (0.51,0.81)	
Q3 (0, [-1, 0])	114/492	0.69 (0.53, 0.89)		0.69 (0.52, 0.90)		0.54 (0.40,0.72)	
Q4 (1, [> 0])	143/564	0.77 (0.61, 0.99)		0.76 (0.59, 0.99)		0.48 (0.36,0.64)	

Abbreviation: CAC = Coronary artery calcification, OR = Odds ratio, Ref = Reference, CVH = Cardiovascular health

Model 1: Unadjusted

Model 2: Adjusted by age, gender, race, education

Model 3: Adjusted by age, gender, race, education, current drinker, history of hypertension, history of diabetes, and baseline CVH score.

Supplementary Table 2. Association between CVH changes and abnormal IMT (n=2691)

		Model 1		Model 2		Model 3	
	n/N	OR (95%CI)	p for trend	OR (95%CI)	p for trend	OR (95%CI)	p for trend
Q1 (-4, [<-3])	40/749	Ref	0.027	Ref	0.068	Ref	0.002
Q2 (-1, [-3, -1])	31/922	0.62<br>(0.38, 1.00)		0.63<br>(0.39, 1.03)		0.54<br>(0.33, 0.89)	
Q3 (0, [-1, 0])	11/470	0.42<br>(0.22, 0.84)		0.47<br>(0.24, 0.93)		0.35<br>(0.17, 0.72)	
Q4 (1, [> 0])	16/550	0.53<br>(0.29, 0.96)		0.57<br>(0.31, 1.04)		0.35<br>(0.18, 0.67)	

Abbreviation: IMT = Intima-media thickness, OR = Odds ratio, Ref = Reference, CVH = Cardiovascular health

Model 1: Unadjusted

Model 2: Adjusted by age, gender, race, education

Model 3: Adjusted by age, gender, race, education, current drinker, history of hypertension, history of diabetes, and baseline CVH score.

Supplementary Table 3. Linear association between CVH changes and IMT (n=2691)

	Model 1		Model 2		Model 3	
IMT	Beta (se)	p-value	Beta (se)	p-value	Beta (se)	p-value
Changes of CVH						
(Per 1 point in the score)	-0.008 (0.001)	<0.001	-0.007 (0.001)	<0.001	-0.012 (0.001)	<0.001

Abbreviation: IMT = Intima-media thickness, OR = Odds ratio, CVH = Cardiovascular health

Model 1: Unadjusted

Model 2: Adjusted by age, gender, race, education

Model 3: Adjusted by age, gender, race, education, current drinker, history of hypertension, history of diabetes, and baseline CVH score.

Supplementary Table 4. Changes from different baseline CVH Class with Prevalence of abnormal IMT (n=2691)

		Model 1		Model 2		Model 3	
IMT	n/N	OR	p-value	OR	p-value	OR	p-value
Change from low class	11/148						
Low to Low	10/92	Ref		Ref		Ref	
Low to Moderate/High	1/56	0.15 (0.02,1.20)	0.073	0.16 (0.02,1.30)	0.086	0.13 (0.01,1.17)	0.068
Change from moderate class	72/1500						
Moderate to Moderate	32/908	Ref		Ref		Ref	
Moderate to Low	37/430	2.60 (1.58,4.20)	<0.001	2.52 (1.52,4.12)	<0.001	2.46 (1.46,4.12)	0.001
Moderate to High	3/162	0.52 (0.16,1.71)	0.279	0.63 (0.19,2.14)	0.463	0.70 (0.20,2.37)	0.564
Change from High class	15/1043						
High to High	6/441	Ref		Ref		Ref	
High to low	1/58	1.27 (0.15,10.76)	0.825	0.58 (0.06,5.32)	0.634	0.60 (0.06,5.64)	0.659
High to moderate	8/544	1.08 (0.37,3.14)	0.885	0.67 (0.21,2.05)	0.468	0.76 (0.24,2.41)	0.642

Abbreviation: IMT = Intima-media thickness, OR = Odds ratio, Ref = Reference, CVH = Cardiovascular health

Model 1: Unadjusted

Model 2: Adjusted by age, gender, race, education

Model 3: Adjusted by age, gender, race, education, current drinker, history of hypertension, history of diabetes, and baseline CVH score

Supplementary Tables 5. Characteristics for follow-up and lost to follow-up participants at baseline

	Lost to follow-up n=2163	Follow-up n=2935	p-value
Age, years	24.4 (3.65)	25.0 (3.59)	<0.001
Male	1038 (48.0%)	1285 (43.8%)	0.003
Black	1270 (58.7%)	1372 (46.7%)	<0.001
Education level	13.3 (1.95)	13.9 (1.99)	<0.001
Smoking Status			<0.001
Never	799 (37.0%)	750 (25.6%)	
Ever	125 (5.78%)	168 (5.72%)	
Current	1237 (57.2%)	2017 (68.7%)	
Alcohol consumption			0.711
Never	190 (8.78%)	248 (8.45%)	
Current/Ever	1973 (91.2%)	2687 (91.6%)	
BMI, kg/m^2	24.7 (5.08)	24.2 (4.59)	0.001
Physical activity	417 (302)	422 (300)	0.602
SBP, mmHg	111 (11.2)	110 (10.7)	<0.001
DBP, mmHg	68.8 (9.99)	68.5 (9.35)	0.287
Fasting glucose, mg/dL	83.3 (20.8)	82.1 (12.2)	0.021
Total cholesterol, mg/dL	176 (34.3)	177 (32.9)	0.277
History of hypertension	201 (9.57%)	262 (8.93%)	0.468
History of diabetes	22 (1.04%)	21 (0.72%)	0.278
Medicine for lowering blood pressure	55 (2.55%)	60 (2.05%)	0.275
Medicine for lowering high cholesterol	0 (0.00%)	1 (0.18%)	1.000
Medicine for lowering blood glucose	5 (1.19%)	4 (0.70%)	0.506

Abbreviation: BMI = Body mass index, SBP = Systolic blood pressure, DBP = Diastolic blood pressure

Supplementary Tables 6. Baseline and Changes of Cardiovascular health with Prevalence of CAC and IMT

Model 4	CAC		IMT	
	OR (95%CI)	p-value	OR (95%CI)	p-value
Baseline				
Per 1 point increase	0.82 (0.78,0.86)	<0.001	0.83 (0.75, 0.93)	0.001
Changes of CVH				
Per 1 point increase	0.86 (0.82, 0.90)	<0.001	0.81 (0.73, 0.90)	<0.001

Abbreviation: CAC = Coronary artery calcification, OR = Odds ratio, CVH = Cardiovascular health, IMT = Intima-media thickness

Model 4: Adjusted by age, gender, race, education, current drinker, history of hypertension, history of diabetes, and family income at Y5.

* In analysis of changes of CVH, Model 4 was adjusted both by baseline CVH score.

Subgroup	n/N	OR (95%CI)	p−value	p for interaction
**Gender**				**0.511**
Male	493/1220	0.89 (0.83, 0.94)	<0.001	
Female	234/1592	0.83 (0.77, 0.90)	<0.001	
**Race**				**0.912**
Black	295/1307	0.86 (0.80, 0.92)	<0.001	
White	449/1505	0.86 (0.81, 0.92)	<0.001	
**History of hypertension**				**0.216**
No	477/2272	0.86 (0.82, 0.91)	<0.001	
Yes	98/253	0.77 (0.66, 0.90)	0.001	
**History of diabetes**				**0.941**
No	734/2793	0.86 (0.82, 0.90)	<0.001	
Yes	10/19	–	–	
**Alcohol Use**				**0.014**
No	100/385	0.97 (0.85, 1.12)	0.696	
Yes	644/2427	0.84 (0.80, 0.89)	<0.001	

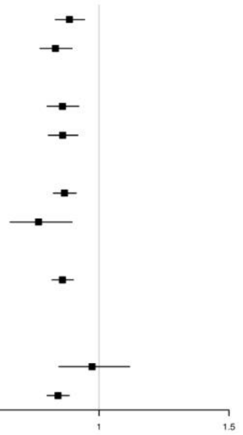

Supplementary Figure 1. Subgroup analysis for CAC

Subgroup	n/N	OR (95%CI)	p−value	p for interaction
**Gender**				**0.463**
Male	65/1175	0.83 (0.73, 0.95)	0.006	
Female	33/1516	0.80 (0.66, 0.96)	0.015	
**Race**				**0.977**
Black	50/1240	0.86 (0.74, 1)	0.051	
White	48/1451	0.78 (0.67, 0.91)	0.002	
**History of hypertension**				**0.983**
No	85/2455	0.81 (0.72, 0.91)	<0.001	
Yes	13/236	0.82 (0.60, 1.13)	0.22	
**History of diabetes**				**0.908**
No	96/2672	0.81 (0.73, 0.90)	<0.001	
Yes	2/19	–	–	
**Alcohol Use**				**0.082**
No	18/369	1.02 (0.79, 1.31)	0.877	
Yes	80/2322	0.77 (0.68, 0.87)	<0.001	

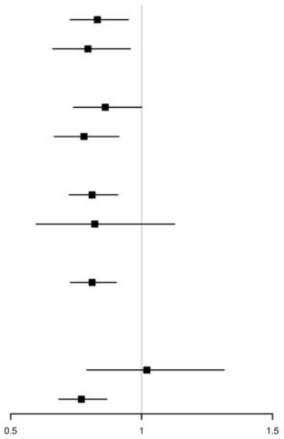

Supplementary Figure 2 Subgroup analysis for IMT

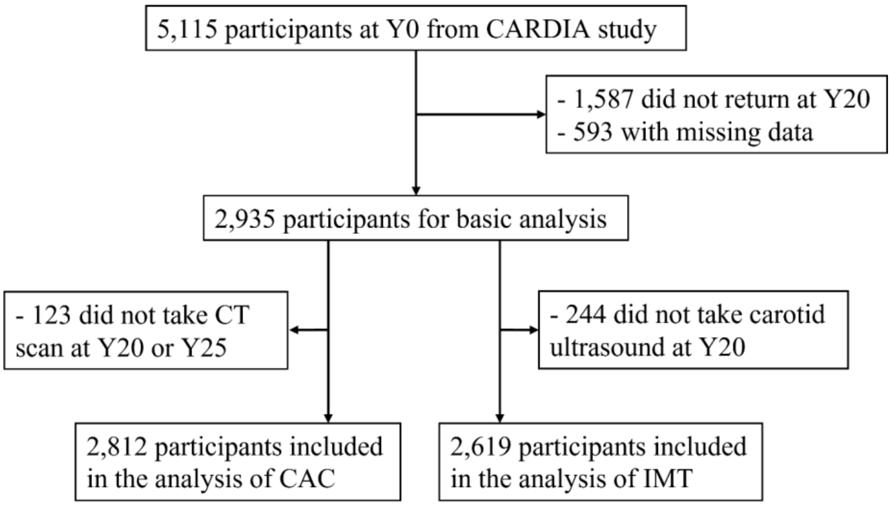

Supplement figure 3. Flow chart

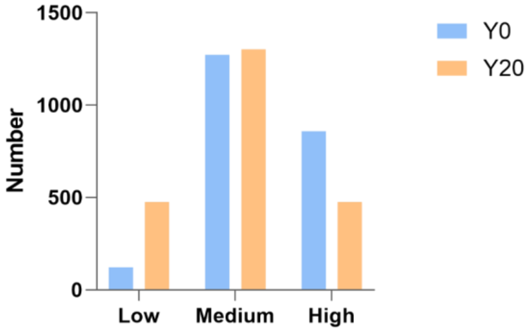

Supplemeantary figure 4. Cardiovascular health class

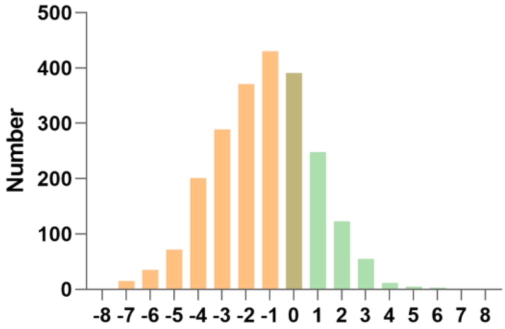

Supplementary figure 5. 20-year changes of Cardiovascular health scores

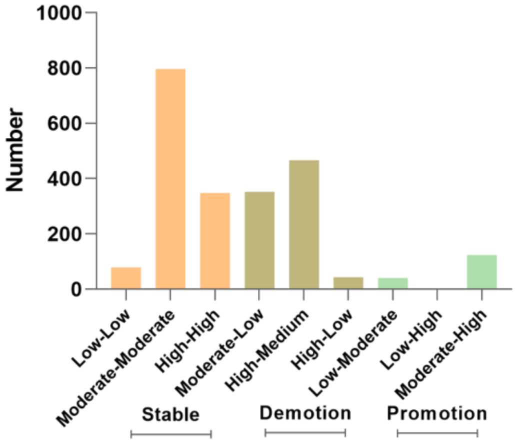

Supplementary figure 6. 20-year changes of Cardiovascular health scores
